# *In Vitro* Anticariogenic Effects of *Drymocallis rupestris* Extracts and Their Quality Evaluation by HPLC-DAD-MS^3^ Analysis

**DOI:** 10.3390/molecules18089117

**Published:** 2013-07-30

**Authors:** Michał Tomczyk, Małgorzata Pleszczyńska, Adrian Wiater, Sebastian Granica

**Affiliations:** 1 Department of Pharmacognosy, Faculty of Pharmacy, Medical University of Białystok, ul. Mickiewicza 2a, 15-230 Białystok, Poland; 2 Department of Industrial Microbiology, Institute of Microbiology and Biotechnology, Maria Curie-Skłodowska University, ul. Akademicka 19, 20-033 Lublin, Poland; E-Mails: mplesz@poczta.onet.pl (M.P.); adrianw2@o2.pl (A.W.); 3 Department of Pharmacognosy and Molecular Basis of Phytotherapy, Faculty of Pharmacy, Medical University of Warsaw, ul. Banacha 1, 02-097 Warsaw, Poland; E-Mail: sgranica@wum.edu.pl

**Keywords:** *Drymocallis rupestris* (Rosaceae), extracts, polyphenolic compounds, anticariogenic activity

## Abstract

In this study, for the first time, we investigated *in vitro* inhibitory effects of *Drymocallis rupestris* extracts and their subfractions obtained with solvents of different polarity (aqueous, 50% ethanolic, diethyl ether, ethyl acetate and *n*-butanolic) against bacterial viability and caries virulence factors of *Streptococcus* spp. strains. The diethyl ether subfraction (PRU2) showed bacteriostatic and bactericidal activity against mutans streptococci, with minimum inhibitory concentrations (MICs) in the range of 0.75–1.5 mg/mL and minimum bactericidal concentrations (MBCs) in the range of 1.5–3 mg/mL. Furthermore, PRU2 inhibited biofilm formation by *Streptococci* in a dose-dependent manner. It was also found that all five *D. rupestris* preparations exhibited diverse inhibitory effects on *de novo* synthesis of water-insoluble and water-soluble α-d-glucans by glucosyltransferases of the mutans group streptococci. The phytochemical profile of investigated samples was determined by spectrophotometric and chromatographic (HPLC-DAD-MS^3^) methods. The high polyphenol (total phenol, phenolic acids, tannins, proantocyanidins, and flavonoids) contents were found which correlated with anticariogenic activity of the analyzed samples. The results demonstrate that *D. rupestris* extracts and their subfractions could become useful supplements for pharmaceutical products as a new anticariogenic agent in a wide range of oral care products. Further studies are necessary to clarify which phytoconstituents of *D. rupestris* are responsible for anticaries properties.

## 1. Introduction

Rock cinquefoil [*Drymocallis rupestris* (L.) Soják (syn. *Potentilla rupestris* L.; *Potentilla corsica* Sieber ex Lehm) Rosaceae] is chiefly found on soils derived from base-rich, igneous rocks. On the European continent, *D. rupestris* is usually found in mountain areas on rock and scrubby, rocky slopes which are often steep and south-facing [1,2]. However, little is known about chemical composition of extracts prepared from *D. rupestris*. The plant has only been reported to contain 2-pyrone-4,6-dicarboxylic acid as well as long-chain polyprenyl alcohols. More recently, the presence of flavonoids and carboxylic acids in the aerial parts of rock cinquefoil has also been described [3,4,5].

Considering the traditional use of the *Potentilla* L. species [6], it is justified to investigate its biological properties in the dental field. Dental caries is a common chronic, social disease, and therefore is an important issue not only for dentists, but also researchers from other fields of science. There is a whole range of methods for preventing or reducing tooth decay. These include, among the others: proper oral hygiene (brushing, toothpaste, dental floss, and mouthwash); the use of unfermentable sugar substitutes [7]; addition of fluoride to drinking water, salt, milk or toothpaste [8]; the use of antimicrobial agents that inhibit the development of dental plaque [9]; sealing of occlusal pits and fissures of caries-susceptible teeth [10]; the induction of immune responses [11], and replacement therapy [12]. However, the problem of caries still remains valid. Hence searching for alternative prevention methods and products for oral diseases that are safe, effective and economical is needed. One of them is the use of plant-derived products [13,14].

The purpose of this study was to evaluate the effects of extracts and their subfractions prepared by the partitioning of aqueous methanolic extract with solvents of different polarity against cariogenic *Streptococcus* spp. strains. Furthermore, their inhibitory effects on *de novo* synthesis of water-soluble and water-insoluble α-glucans by glucosyltransferases of mutans streptococci and artificial dental plaque formation were examined. The phytochemical profile of the investigated samples was also determined.

## 2. Results and Discussion

### 2.1. Determination of Polyphenolic Compounds

In order to roughly estimate the total polyphenol content (TPC), and that of related polyphenolic compounds like phenolic acids (TPA), flavonoids (TFC), tannins (TTC), proanthocyanidins (TPDC) in the aerial parts of *D. rupestris* (Table 1) were measured by different analytical methods preceeding the HPLC-MS study. The results obtained in the present study showed that all the analyzed extracts and subfractions contain relatively huge quantities of polyphenolic compounds. Both, the diethyl ether subfraction (PRU2) and the ethyl acetate subfraction (PRU3) showed the highest values of the total phenolic content (Table 1), which varied from 29.9 ± 0.7 mg GAE/g dw for PRU2 to 30.9 ± 1.1 mg GAE/g dw for PRU3. It was obvious that the total phenolic content determined by Folin-Ciocalteu’s method had not given a full characterization of the quality and quantity of the various groups of polyphenolics. The presence of different groups of phenolic compounds in investigated plant material was determined by weight and spectrophotometrical methods. The results (Table 2) obtained showed that aerial parts of *D. rupestris* contained relatively high quantities of tannins (115.0 ± 3.7 mg/g dw) proanthocyanidins (4.6 ± 0.4 mg/g dw) and phenolic acids (7.8 ± 0.4 mg/g dw), as well as flavonoids (4.2 ± 0.3 mg/g dw) and (6.0 ± 0.5 mg/g dw), calculated as quercetin, flavonol type compound and parallel as a glycoside hyperoside, respectively.

**Table 1:** Total phenolic content in the obtained extracts and their subfractions from *D. rupestris*.

Extracts	Total phenolic content (TPC) (mg GAE/g dry weight) ^a^
PRU	26.7 ± 0.8
PRU1	23.2 ± 0.5
PRU2	29.9 ± 0.7
PRU3	30.9 ± 1.1
PRU4	22.5 ± 0.9

^a^ all data are shown as means ± SD of at least three independent experiments.

**Table 2:** Content of polyphenolic compounds in *D. rupestris*

Polyphenols content (mg/g dry weight) ^a^				
Phenolic acids (TPA)	Flavonoids (TFC)		Tannins (TTC)	Proanthocyanidins (TPDC)
	Quercetin	Hyperoside		
7.8 ± 0.4	4.2 ± 0.3	6.0 ± 0.5	115.0 ± 3.7	4.6 ± 0.4

^a^ results are means ± SD of three different experiments.

**Figure 1:**
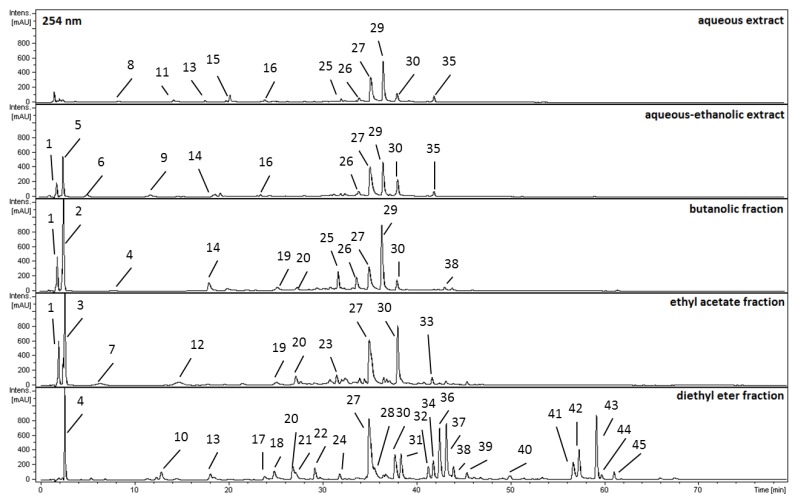
HPLC-DAD-MS^3^ total ion chromatogram of analyzed extracts and their subfractions acquired at 254 nm. For chromatographic conditions see Experimental section. Numbering of the peaks is the same as in Table 3

It is noteworthy that according to our previous observations these differences in the values of total phenolic content (TPC) between all analyzed extracts and their subfractions can be attributed to the differences in their phytochemical composition. The total tannin content (TTC) determined was higher that this reported as the sum of all polyphenolic compounds (TPC). The significant difference found between this value arise from the use of two different analytical methods [15,16]. The observed significant differences between the total content of tannins (TTC) (155 mg/g) and condensed tannins (TPDC) (4.6 mg/g) in spectrophotometric assays was additionally confirmed by using the HPLC-DAD-MS^3^ method. In fact, the hydrolysable tannins are predominant compounds both in the aerial parts as well as in the extracts from this plant. Forty five chemicals, numbered as 1–45, were detected (Figure 1) and tentatively assigned as belonging to phenolic acid, tannin as well as flavonoid derivatives. The identification (Table 3) of all the compounds was carried based on comparison of their retention times, UV-Vis and MS spectra with chemical standards available or by comparison of UV-Vis, MS and MS/MS spectra with those found in literature.

### 2.2. Anticariogenic activity

The effects of *D. rupestris* extracts and their subfractions on planktonic growth of *Streptococci* cells are shown in Table 4. The minimum inhibitory concentration (MIC) and minimum bactericidal concentration (MBC) were determined based on micro-dilution method. Among the samples tested, the diethyl ether subfraction (PRU2) exhibited the significant bacteriostatic and bactericidal activity against all the tested bacteria with MICs of 0.75 or 1.5 mg/mL and MBCs of 1.5 or 3 mg/mL (see Table 4).

**Figure 2:**
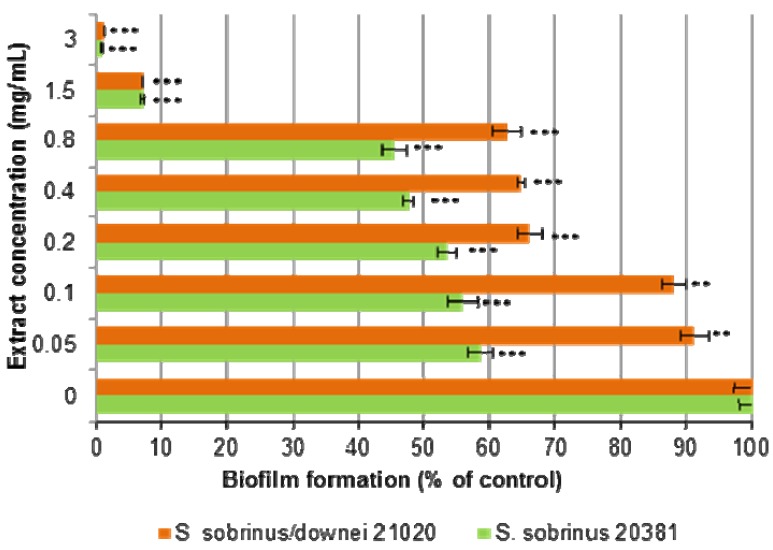
The inhibitory effects of diethyl fraction (PRU2) on mutans streptococci biofilm formation. The relative amount of biofilm formation, at a certain PRU2 concentration, is given compared to non-treated controls. Chlorhexidine, used as a positive control at sub MIC concentration (2.5 mg/L), showed 93.5 ± 0.6 and 95 ± 0.8% inhibition on *S. sobrinus/downei* 21020 and *S. sobrinus* 20381 biofilm formation, respectively. Results are means ± SD of three different experiments (n = 3), * *p* < 0.05, ** *p* < 0.01, *** *p* < 0.005.

The effect of PRU2 on biofilm formation by the two selected strains, *S. sobrinus* 20381 and *S. sobrinus/downei* 21020, was examined by method similar for MIC assays for planktonic cells. The growth of *S. sobrinus* 20381 in a biofilm demonstrated higher susceptibility for PRU2 extract than the growth of *S. sobrinus/downei* 21020 (Figure 2).

However, the biofilm formation was almost completely inhibited at PRU2 concentration of 3 mg/mL. Thus, it was found that *Streptococci* cells in biofilm were somewhat more resistant to the diethyl ether subfraction compared to their planktonic form. It is probably related to higher biomass densities and decreased metabolic activities in biofilm, which affect the effectiveness of the therapeutic agent. 

**Table 3:** HPLC-DAD-MS identification of compounds contained in *D. rupestris*.

	Identified compounds	Retention time [min]	UV [nm]	[M-H]^−^ *m/z*	MS^2^ ions	MS^3^ ions	[M+H]^+^	MS^2^ ions	MS^3^ ions
1	HHDP-glucose	2.1	260	481	421, 301b	-	-	-	-
2	unknown compound	2.6	220, 258	639	613, 463b, 301	-	641	-	-
3	unknown tannin	2.7	257	783	-	-	785	-	-
4	gallic acid *	2.7	266	169	125b	-	171	127b	-
5	unknown compound	2.8	262	447	315b, 297, 207, 177, 163	-	-	-	-
6	unknown tannin	5.4	248	391^a^, 783	481, 421, 301b, 275	-	785	767b, 465, 303	-
7	unknown tannin	6.7	249	391^a^, 783	481, 421, 301b, 275	-	785	767b, 465, 303	-
8	bis-HHDP-glucose isomer	9.0	216, 260 sh	783	481, 421, 301b	-	785	767b, 465, 303	-
9	bis-HHDP-glucose isomer	12.2	250	783	481, 301b, 275	-	-	-	-
10	methyl gallate *	13.0	271	183	167b	-	185	153b	-
11	bis-HHDP-glucose isomer	14.8	216, 260 sh	783	481, 421, 301b	-	785	767b, 465, 303	-
12	galloyl-HHDP-glucose isomer	15.2	271	633	463, 301b	-	657^b^	-	-
13	brevifolincarboxilic acid	18.1	245, 296, 324	291	247b, 279	-	293	257, 169	-
14	quercetin derivative	18.3	260, 350	757	595b	463, 445, 343, 301b	759	627, 465b, 303	465b, 303
15	quercetin glucuronylhexoside	19.2	273, 353	639	463b, 301	343, 301b	641	465b, 303	303b
16	caffeoylquinic acid isomer	23.8	234 sh, 303 sh, 327	353	191, 173b, 155, 127, 111	-	377^b^	243, 215b, 197, 185	-
17	ellagic acid derivative	24.0	255, 261	469	451, 425b, 301, 167	-	493^b^	475b, 452, 409, 333	-
18	unknown flavonoid	25.1	259, 295 sh, 357	399	273b	-	401	275b, 247	-
19	ellagic acid 3,3'-di-*O*-methyl ether 4-*O*-xyloside *	25.2	250, 325	461	415b, 301	-	463	445, 331b, 275	271, 257b, 169
20	unknown compound	27.3	274	247	219, 191b	-	249	207b, 186	-
21	unknown compound	27.4	224 sh, 266, 302	275	257b, 229, 203	-	277	259b, 249, 215	-
22									
23	unknown flavonoid	31.6	270, 350	639	459b, 444, 315	-	641	479, 317b	461, 413, 317b
24	unknown compound	31.9	270, 360	365	183b, 153	-	367	335b, 303	-
25	quercetin pentosylglucuronide	32.5	258, 351	609	301b, 179	-	633^b^, 611	597, 479b, 303	303
26	unknown flavonoid	34.6	250, 351	447	369, 301b	-	449	303b	285b, 275, 258
27	ellagic acid *	35.5	253, 362	301	284, 257b, 185	-	303	285b, 248	-
28	uknown compound	35.9	slope	435	303b, 285, 177	285b, 177	459^b^	441, 412, 356, 327b, 307, 251	-
29	quercetin 3-*O*-glucuronide *	37.0	252, 350	477	301b	273, 179b, 151	479	303b	285, 257b, 227, 165
30	unknown compound	38.1	226, 292	435	303b, 285, 177	285b, 177, 125	-	-	-
31	unknown compound	38.5	264, 290 sh, 350	457	273b, 245	-	459	427b, 275, 261	-
32	quercetin 3-*O*-arabinoside *	41.4	267, 351	433	301b	-	435	303b	285, 275, 257b, 207, 165
33	unknown flavonoid pentoside	41.7	250, 361	447	315b, 300	299	449	317b, 286	286
34	kaempferol 3-O-glucoside *	41.9	265, 340	447	327, 299, 285b, 255	-	449	287b	269, 241, 213, 153, 121b
35	kaempferol 3-*O*-glucuronide *	42.5	265, 348	461	285b	267, 257b, 241, 229, 213, 197, 185	463	287b	241, 213, 127b
36	ellagic acid derivative	42.6	255, 366	483	451b, 407, 315, 301	-	485	471, 453b, 435	-
37	valoneic acid dilactone methyl ester *	43.3	255, 366	483	451b, 407, 315, 301	-	485	471, 453b, 435	-
38	isorhamnetin glucuronide	44.1	257, 265 sh, 355	491	473, 315b, 301	300b, 287, 272	493	303b	-
39	ellagic acid derivative	45.3	257, 355	457	275b	-	459	427, 409b, 399, 257	-
40	unknown compound	50.0	267, 315	445	265, 235, 205, 163, 145b	-	469^b^	377, 306, 173, 147b	-
41	quercetin*	56.7	255, 368	301	273, 257, 229, 179b, 151	-	303	275b, 257, 230, 165	-
42	unknown compound	57.4	253, 267	483	251b, 301	-	485	453b	-
43	tiliroside*	59.3	267, 311, 354 sh	593	447, 285b	285	595	329, 309, 287b, 165	-
44	unknown flavonoid	59.9	267, 315, 360 sh	623	461, 447, 332b, 299, 285	341, 299, 285	625	339b, 321, 287, 177	-
45	tiliroside isomer	61.1	267, 308, 351 sh	593	447, 307, 285b	327, 285b, 255	595	392, 309, 287b	-

^a^ [M-2H]^2−^; ^b^ [M+Na]^+^; * comparisons with chemical standard have been made; sh — shoulder.

**Table 4:** MIC and MBC values of *D. rupestris* samples against mutans streptococci

Tested strains	MIC (mg/mL)						MBC (mg/mL)					
	PRU	PRU1	PRU2	PRU3	PRU4	CHX ^a^	PRU	PRU1	PRU2	PRU3	PRU4	CHX ^a^
*S. sobrinus/downei* 21020	>12	>12	1.5	>12	>12	3.125 × 10^−3^	>12	>12	1.5	>12	>12	6.25 × 10^−3^
*S. sobrinus* DSM 20381	>12	>12	0.75	>12	>12	3.125×10^−3^	>12	>12	1.5	>12	>12	3.125×10^−3^
*S. mutans* 6067	6	6	1.5	6	>12	3.125 × 10^−3^	12	12	3	12	>12	3.125×10^−3^
*S. sobrinus* 6070	>12	6	1.5	6	>12	3.125 × 10^−3^	>12	12	3	12	>12	6.25×10^−3^
*S. sanguis* ATCC 10556	>12	3	1.5	3	>12	3.125 × 10^−3^	>12	6	3	6	>12	6.25×10^−3^

^a^ CHX - chlorhexidine digluconate (Sigma-Aldrich) final concentration from 25 to 0.08 µg/mL, was used as positive reference standard.

The structure of polysaccharides contained in the dental plaque matrix is the key factor in its ability to cause tooth decay, as it exerts an effect on the physical and biochemical properties of the biofilm. Matrix polysaccharides can increase microbial adhesion and biofilm cohesion, serve as an additional source of energy, protect microorganisms from hostile interactions, affect diffusion of substances to and from the biofilm, and facilitate concentration of ions of metals and other important nutrients in the biofilm environment. Among the great number of polymers synthesized by glucosyltransferases of cariogenic streptococci (1→6-α-d-, 1→4-α-d- and 1→3-α-d-glucans), the water-insoluble (1→3)-α-d-glucan plays a fundamental role in the etiology of dental caries because of unique properties that contribute to the formation of the plaque skeleton, *i.e.*, it is easily adsorbed to saliva- or dental pellicle-coated enamel, promotes bacterial co-aggregation, and substantially enhances cohesion of plaque [17].

**Figure 3:**
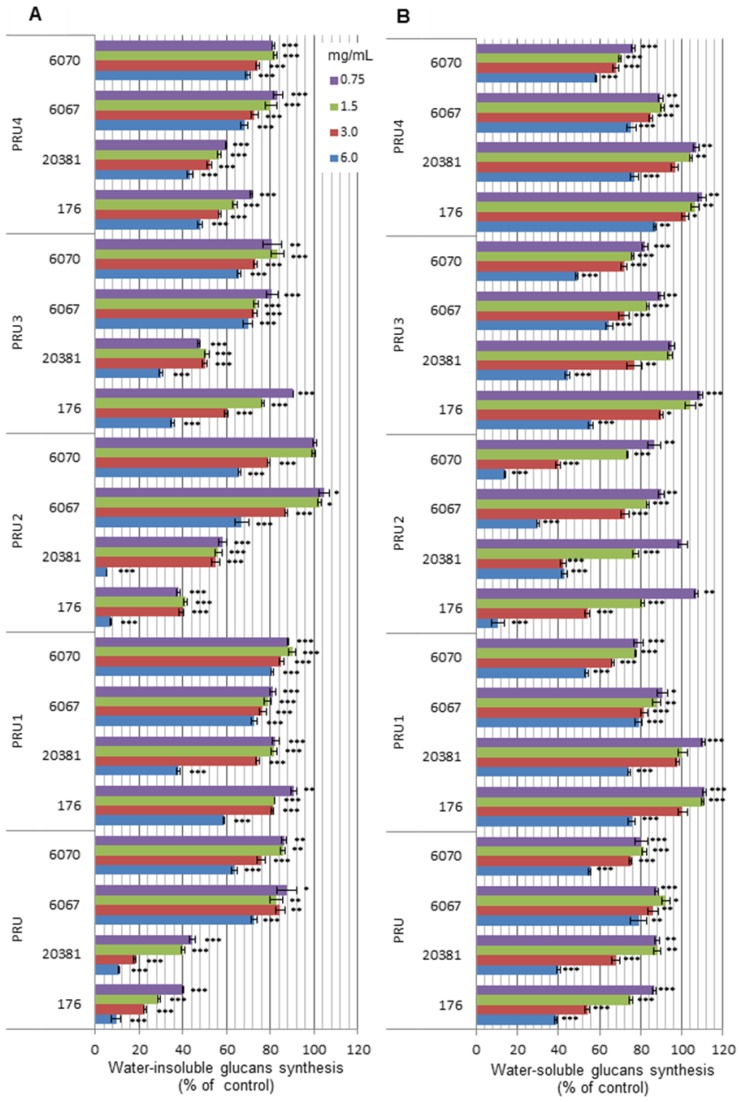
Inhibitory effects of *D. rupestris* preparations on water-insoluble (**A**) and water-soluble (**B**) glucan synthesis by glucosyltransferases of mutans streptococci. Results are means ± SD of three different experiments (n = 3), * *p* < 0.05, ** *p* < 0.01, *** *p* < 0.005.

The effects of all tested preparations of *D. rupestris* on *de novo* synthesis of water-insoluble (IG) and water-soluble (SG) glucan by cell-free GTFs isolated from *Streptococcus* spp. were examined as a function of their concentrations and were expressed as the relative amount (%) of glucans produced at the certain extract or subfraction concentration compared to the amount produced in the absence of any plant substance (Figure 3A and B).

It was found that all five formulations affected the synthesis of both types of glucans but to different degrees depending on preparation’s concentration and streptococcal strain using as a GTFs source. In general, the degree of inhibition was proportional to the increasing concentrations of preparations, the effect of phytochemicals was more pronounced in the case of water-insoluble polymers.

Glucan-synthesis activity of cell-free GTFs of *S. sobrinus/downei* 21020 and *S. sobrinus* 20381 was more strongly suppressed than the activity of GTFs of the other two strains. Both, PRU and PRU2 fractions showed the highest activity. A significant (60%) reduction of IG synthesis by *S. sobrinus/downei* 21020 was found at 0.75 mg/mL of PRU or PRU2. In the case of SG, the same effect was achieved only at a concentration of 6.0 mg/mL. 

## 3. Experimental

### 3.1. Plant material

Seeds of *D. rupestris* (index seminum 763) were requested from the Giardino Botanico Alpino, Cogne, Italy. Plants were cultivated in a common plots at the Medicinal Plants Garden near Medical University of Białystok, Poland. Aerial parts of plants were collected during June–August 2008–2010. Voucher specimen of plant PRU-06021 has been deposited in the Herbarium of the Department of Pharmacognosy, Medical University of Białystok, Poland.

### 3.2. Preparation of Extracts and Their Subfractions

Different solvent systems (water, 50% ethanol, diethyl ether, ethyl acetate and *n*-butanol) were used to prepare the extracts and subfractions. Powdered plant material (2.0 g) was separately extracted with water (2 × 150 mL) – PRU or 50% ethanol (2 × 150 mL) – PRU1 in an ultrasonic bath (Sonic-5, POLSONIC, Warsaw, Poland) at controlled temperature (40 ± 2 °C) for 45 min. Supernatants were filtered through a funnel with glass wool, which was washed with 5 mL of solvent and concentrated to dryness under vacuum (Büchi Labortechnik System, Flawil, Switzerland) at controlled temperature (40 ± 2 °C), suspended in water and subjected to lyophilization using LABCONCO vacuum concentrator until a constant weight was obtained. Yields: PRU–79 mg; PRU1–92 mg.

Accurately weighed powdered plant material (2.0 g) was separately extracted with methanol (3 × 50 mL) and once with 50 mL of 80% (v/v) methanol in an ultrasonic bath (Sonic-5, POLSONIC) at controlled temperature (40 ± 2 °C) for 45 min. After solvent evaporation under reduced pressure from each sample, the methanolic extracts were diluted with water and successively partitioned between chloroform and then diethyl ether (PRU2), ethyl acetate (PRU3) and *n*-butanol (PRU4). All subfractions were concentrated to dryness under vacuum controlled temperature (Büchi) (temperature: 40 ± 2°C), suspended in water and subjected to lyophilization using a LABCONCO vacuum concentrator until a constant weight was obtained. Yields: PRU2—16 mg; PRU3—36 mg; PRU4—103 mg.

### 3.3. Phytochemical Profile

#### 3.3.1. Determination of Total Polyphenol Content

Total polyphenol content in extracts was determined at 765 nm (SPECORD 40, Analytik Jena, Jena, Germany) after the reaction with Folin-Ciocalteu’s phenol reagent as gallic acid equivalents GAE/100g in mg per g of dry weight (dw) according to the manual colorimetric method described by Tawaha [18]. A sample aliquot of extract or subfraction (50 μL) was dissolved in distilled water (450 μL) and 0.2 N Folin-Ciocalteu’s reagent (2.5 mL). After 5 min saturated sodium carbonate Na_2_CO_3_ solution (75 g/L, 2 mL) was added. Samples were vortexed and incubated in the darkness at room temperature for 2 h. Quantitative measurements were performed, based on a standard calibration curve of different concentrations of gallic acid (20–500 mg/L). All measurements were performed in triplicate. The results are given in Table 2.

#### 3.3.2. Determination of Total Phenolic Acids Content

Total phenolic acids content in plant material was determined by use the spectrophotometric method with Arnov’s reagent according to the procedure described in Polish Pharmacopoeia IX [19]. Stock solution was prepared from the powdered sample (1.0 g) mixed with water (25 mL, two times) and shaken for 30 min each, then filtered. Phenolic acids were determined from a stock solution aliquot (1 mL) mixed with water (5 mL), hydrochloric acid (18 g/L, 1 mL), Arnov’s reagent (1 mL) and sodium hydroxide solution (40 g/L, 1 mL) and diluted with water to 10 mL. Phenolic acids were measured spectrophotometrically at 490 nm. The percentage of phenolic acids, expressed as caffeic acid equivalents on dry weight, is calculated according to the formula:

*A* × 1.7544/*m* (1)

where *A* is the absorbance of the test solution at 490 nm and *m* mass of the powdered drug, in grams. The results are given in Table 2 as means of experiments conducted in triplicate.

#### 3.3.3. Determination of Total Flavonoid Content

The total content of flavonoids was determined by the spectrophotometric method by Christ and Müller [20] and followed the procedure described in Polish Pharmacopoeia IX [19]. Each powdered sample (0.6 g) was mixed with acetone (20 mL), 25% hydrochloric acid (281 g/L, 2 mL) and 0.5% urotropine (methenamine) solution (5 g/L, 1 mL) and heated in a water bath under reflux for 30 min. The obtained extract was filtered through cotton wool, and the sediment with the cotton wool was heated twice for 10 min with acetone (20 mL). The extracts were mixed and diluted with acetone to 100 mL in a volumetric flask. Then, this solution (20 mL) was diluted with water (20 mL), extracted with ethyl acetate (15 mL) and then, three time with ethyl acetate (10 mL). Organic phases were mixed and washed twice with water (40 mL), filtered into a volumetric flask and diluted with ethyl acetate to 50 mL. The obtained solution (10 mL) was added to four volumetric flasks. Then, to three flasks aluminum chloride (20 g/L – methanolic solution, 2 mL) was added and all four flask were filled with methanol–acetic aid glacial (19:1) to 25 mL. After hydrolysis, the flavonoids were measured spectrophotometrically at 425 nm by creating a complex with aluminum chloride in a methanol–ethyl acetate–acetic acid medium. The contents of total flavonoids, expressed as quercetin equivalent on dry weight, according to the formula , respectively:

*A* × 0.875/*b* (2)

where *A* is the absorbance of the test solution at 425 nm and *b* the mass of the powdered drug, in grams. The results are given in Table 2.

#### 3.3.4. Determination of Total Tannin Content

The total tannin content was determined by the weight method with hide powder according to the DAB 10 [21]. The results are given in Table 2.

#### 3.3.5. Determination of Total Proanthocyanidin Content

The total proantocyanidin content was measured according to the European Pharmacopoeia [22]. Accurately weighted plant material (2.5 g) was heated under reflux for 30 min with ethanol (70% v/v, 30 mL). After that extract was filtered and the residue was flashed with ethanol (70% v/v, 10 mL). Then 25% hydrochloric acid (15 mL) was added with water (10 mL). The solution was heated under reflux for 80 min. After cooling, extract was filtered and filled up with ethanol (70% v/v) to 250 mL. Then the solution (50 mL) was evaporated to about 3 mL and transferred to a separatory funnel with water (15 mL). The solution was then extracted with *n*-butanol (3 x 15 mL, each). The organic layers were mixed and transferred to volumetric flasks and filled up with *n*-butanol to 100 mL. The absorbance was measured spectrophotometrically at 545 nm. The contents of proanthocyanidin, expressed as cyanidin chloride equivalent on dry weight, were calculated according to the formula:

*A* × 500/75 × *m* (3)

respectively, where *A* is the absorbance of the test solution at 545 nm and *m* the mass of the powdered drug, in grams. The results are given in Table 2.

#### 3.3.6. HPLC-DAD-MS^3^ Analysis

The HPLC-DAD-MS^3^ analysis was performed using an UHPLC-3000 RS system (Dionex, Idstein, Germany) equipped with a dual low-pressure gradient pump, an autosampler, a column compartment, a diode array detector, and an AmaZon SL ion trap mass spectrometer with an ESI interface (Bruker Daltonik GmbH, Leipzig, Germany). HPLC analyses of extracts and fractions were carried out on a reversed-phase Zorbax SB-C18, 150 × 2.1 mm, 1.9 µm column (Agilent, Santa Clara, CA, USA). Column temperature was 25 °C. The mobile phase (A) was water/acetonitrile/formic acid (95:5:0.1, v/v/v) and the mobile phase (B) was acetonitrile/formic acid (100:0.1, v/v). A two-step gradient system was used: 0–60 min. 1%–26% B, 60–80 min 26%–50% B. The flow rate was 0.2 mL/min. The column was equilibrated for 10 min between injections. UV spectra were recorded over the range of 200–450 nm, chromatograms were acquired at 254 nm and 350 nm. The LC eluate was introduced directly into the ESI interface without splitting. Compounds were analyzed in negative and positive ion mode. The MS^2^ fragmentation was obtained for two the most abundant ion at the time. The detection of neutral loses was set for the sugars moieties characteristic for glycosides fragmentation (132, 146, 162 and 176 *amu*). In the case of detection of one of the neutral loss masses the MS^3^ fragmentation was performed in order to obtain the fragmentation spectrum of the aglycone moiety. The nebulizer pressure was 40 psi; dry gas flow 9 L/min; dry temperature 300 °C; and capillary voltage 4.5 kV. Analysis was carried out using scan from *m/z* 200 to 2.200.

### 3.4. Anticariogenic Activity

#### 3.4.1. Bacterial Strains

The cariogenic streptococci used in this study included *Streptococcus mutans* CAPM 6067, *S. sobrinus* CAPM 6070 (The Collection of Animal Pathogenic Microorganisms, Brno, Czech Republic), *S. sobrinus* DSM 20381, *S. sanguis* ATCC 10556 and *S. sobrinus*/*downei* CCUG 21020 (The Culture Collection, University of Göteborg, Göteborg, Sweden).

#### 3.4.2. Determination of Minimum Inhibitory Concentration (MIC) and Minimum Bactericidal Concentration (MBC)

The *D. rupestris* preparations were tested for antibacterial activity by the broth dilution method. MIC values were determined in 96-well cell culture plates (Nunc, Roskilde, Denmark). A two-fold serial dilution of each sample in 20% DMSO was prepared. Plates with wells containing 180 μL of BHI medium plus 20 μL of 20% DMSO (100% growth controls) or 180 μL of BHI medium plus 20 μL of each dilution of test extracts (extracts final concentration from 12 to 0.09 mg/mL, DMSO final concentration—2%) were inoculated with a bacterial suspension containing 2 × 10^5^ colony forming units/mL, and cultured for 24 h at 37 °C. MICs were determined as the lowest concentration of test samples that resulted in a complete inhibition of visible growth in the broth. After the determination of MICs, 10 μL aliquots of cultures were taken from wells showing no growth, inoculated into plates containing Mueller-Hinton Agar with 5% Sheep Blood (BBL), and cultured for 1 week. The minimum bactericidal concentrations were determined on the basis of the lowest concentration of the test extracts that kills 99.9% of the tested bacteria. Chlorhexidine digluconate (Sigma-Aldrich, St. Louis, MO, USA, final concentration from 25 to 0.08 µg/mL) was used as positive control to determine the sensitivity of each microbial species tested.

#### 3.4.3. Inhibition of Biofilm Formation

Biofilms were cultivated on glass disks placed in a 24-well microtitre plates. Serial dilutions of each plant preparation in 20% DMSO were prepared and aliquots (50 μL) of each dilution were dispensed in wells of culture plates. Subsequently, 950 μL portions of BHI medium with 1% (w/v) sucrose were added, and 10^5^–10^6^ of tested bacteria were inoculated into each well. Final concentrations of extracts ranged from 12 to 0.2 mg/mL, DMSO final concentration was 1%. The medium without extracts was used as a non-treated control. Chlorhexidine digluconate at 2.5 mg/L was used as a positive control. After incubation for 24 h at 37 °C, the disks were removed and media with unattached to substratum cells were removed by washing with phosphate buffered saline (PBS, pH 7.4). The minimum biofilm eliminating concentrations were determined as the lowest concentration of test samples that resulted in a complete inhibition of biofilm growth. For this purpose, three sets of disks were used to stain of the adhered biofilm with 1 mL of 1% erythrosine B for 5 min, rinsed thoroughly with water, and dried in room temperature. The bound dye was then removed from biofilms with 6 mL of 1 M NaOH. Biofilm formation was quantified by measuring optical density at 525 nm. The percentage of inhibition was calculated using the equation:

(1 − A_525_ of the test/ A_525_ of non-treated control) × 100% (4)

#### 3.4.4. Inhibition of streptococcal α-d-glucans synthesis

Crude preparations of *Streptococcus* spp. GTFs were prepared. Bacteria were grown in BHI at 37 °C for 18 h. Cell suspension was centrifuged (9600 × g, 30 min, 4 °C) and the culture supernatants were the source of crude extracellular GTFs. Supernatants were ultrafiltered and concentrated. To measure the inhibitory effects of extracts from *D. rupestris* on α-d-glucans synthesis we used a reaction mixture containing sucrose (30 mg), diluted GTFs (75 µL), and the dilution of each extract (75 µL, final concentration from 0.5 to 6 mg/mL) in 20% DMSO in a total volume of 1.5 mL of sodium phosphate buffer (pH 6.0) containing sodium azide to a final concentration of 0.05%. The medium without test substance was used as the non-treated control. After incubation for 24 h at 37 °C, the formed water-insoluble α-d-glucans were collected by centrifugation and washed twice with water. The water-soluble α-d-glucans were precipitated from the supernatant by addition of absolute ethanol (3 volume) followed by storage for 30 min at 4 °C and washed twice with 75% ethanol. Both glucans were determined utilizing phenol-sulphuric acid method with glucose as a standard [23].

### 3.5. Data Analysis

Statistical analysis was performed on all the three replicates from each treatment. Data were analyzed with Statistica 10.0. One-way analysis of variance was performed, followed by the Duncan test, for comparison of multiple means. The level of significance was *p* < 0.05.

## 4. Conclusions

The results of the study suggest that preparations from *Drymocallis rupestris* are promising natural products for the prevention of dental caries since they demonstrate antimicrobial activity against mutans streptococci and also inhibit *in vitro* the formation of dental plaque. These preparations contain huge quantities of polyphenols such as tannins, phenolic acids as well as flavonoids. Further studies are necessary to clarify which phytoconstituents from *D. rupestris* are responsible for the observed anticaries properties. 
